# The Influence of Seasonal Period and Match Outcome on External Load in Professional Soccer Players: Analyzing the Effect of Winning and Losing Streaks

**DOI:** 10.3390/s25134090

**Published:** 2025-06-30

**Authors:** José C. Ponce-Bordón, Jorge Polo-Tejada, David Lobo-Triviño, Borja Sanabria-Pino, Javier Raya-González, Alberto Muñoz, Tomás García-Calvo

**Affiliations:** 1 Faculty of Sport Sciences, University of Extremadura, 10003 Caceres, Spain; joponceb@unex.es (J.C.P.-B.); jpolotej99@gmail.com (J.P.-T.); bsanabriapino@gmail.com (B.S.-P.); tgarciac@unex.es (T.G.-C.); 2 Research Group on Sport and Physical Education for Personal and Social Development (GIDEPSO), Faculty of Education Sciences and Psychology, University of Cordoba, 14071 Cordoba, Spain; rayagonzalezjavier@gmail.com; 3 Sport Performance Area, CP Cacereño, 10003 Caceres, Spain; ammpf1@gmail.com

**Keywords:** external load, monitoring, football, tracking technology, performance

## Abstract

**Highlights:**

**What are the main findings?**

- The team accumulated higher weekly training load (TL) after medi-um-performance streaks. Contrarily, weekly training volume was lower after drawn matches than after won matches.
- Although there was not a clear trend on evolution on TL, most of variables de-creased as the season progressed.

**What is the implication of the main finding?**

- Identifying the weeks with higher training demands is crucial for effective load management and preventing injury risk for overreaching.
- Practitioners should reduce the weekly training demands after high- or medi-um-performance streaks or, even, after lost matches since players will try to per-form their maximum efforts on those weeks.

**Abstract:**

The aim of this study was threefold: (i) to analyze the influence of previous match outcome on subsequent weekly training load (TL); (ii) to examine whether accumulated weekly TL varies throughout the season; and (iii) to investigate the influence of performance streaks got during competition on subsequent weekly TL. Twenty-one Spanish male professional soccer players from the same team were involved in the study. Total distance (TD), medium-speed running (MSR, distance 10.8–18.0 km·h^−1^), high-speed running (HSR, >21 km·h^−1^), very high-speed running (VHSR, 18.0–25.2 km·h^−1^), sprinting speed running distance (sprint, >25.2 km·h^−1^), player load (PL), number of accelerations (ACC), and decelerations (DEC) were recorded during training sessions using 10 Hz GPS devices. Previous match outcome, period of the season, and the performance streaks were also considered. Linear mixed models showed that team covered significantly less TD during the week after draw than after win (*p* < 0.05). In addition, most of the variables decreased as the season progressed. Finally, after medium-performance streaks, team covered significantly higher TD compared to high-performance streaks (*p* < 0.05) and low performance streaks (*p* < 0.01). These findings showed that low-performance streaks could reduce weekly external TL.

## 1. Introduction

The inclusion of tracking technology in soccer has allowed strength and conditioning coaches to advance in monitoring player workload, providing detailed insights into each training session and official match [1,2]. Global positioning systems (GPS) favor a precise quantification of the external load (i.e., total distance, high-speed running, number of accelerations, etc.) covered by soccer players across different sessions during the microcycle [3,4,5]. The weekly training load (TL) monitoring is a crucial factor in managing player readiness for competition [6]. For instance, recent research has tried to examine the correct weekly TL distribution to optimize the subsequent performance of soccer players [6]. Also, weekly TL monitoring contributes to ensuring that the physical demands of training sessions align with match physical demands [7]. Therefore, this objective method of TL monitoring facilitates a more individualized approach to player conditioning, optimizing the performance for competition while it could potentially reduce the risk of muscle or overreaching injuries [8,9].

A previous study that systematically reviewed the literature on the contextual-related factors affecting match running in soccer reported that situational (i.e., playing formation, scoreline, congested schedule, etc.) and environmental factors (i.e., temperature and altitude) strongly influence the variability and differences observed in match-running performances from match-to-match [10]. This information is relevant in a match analysis setting for professional match analysts. However, there is scarce published literature examining the influence of context-related variables on weekly TL, with only a few studies addressing this topic [11]. One of these factors is the match outcome from the previous match. In this regard, higher training volume has been observed after losing matches compared to wins in professional male soccer teams [11,12]. Similarly, another recent study found that weekly training volume following a win was lower compared to after a loss or draw [13]. Since what occurs in the previous match can influence the subsequent TL, soccer coaches need to consider the impact of certain context-related variables on training responses when planning training sessions. This awareness allows for better decision-making and a more accurate weekly TL distribution to optimize players’ subsequent performance [6], either by increasing or reducing the TL of the next microcycle depending on contextual demands [12].

Another important factor to consider in the soccer periodization is the seasonal periods. Specifically, in a professional soccer setting, the competition lasts for up to 10 months with official matches every week. So, physical performance may decrease in different periods of the season; however, the competitive schedule implies the need to maintain high levels of performance across the season [13]. Concerning the evolution of match running performance across the season, most studies have reported that during the mid-season period, soccer players reach their best physical output, with higher total distances covered at different speed thresholds and a higher number of meters at maximum speed. For instance, previous research based on match physical demands has established that during the mid-season, the total distance covered (10,318 m) by a professional soccer team was greater than in the early season (9806 m; [14]). Additionally, as the season progressed, players tended to cover greater distances at higher speeds, probably due to the coaches’ still having some emphasis on physical conditioning and the subsequent neuromuscular and physiological adaptations [14]. Similarly, other studies analyzing the 2016/17 Greek Super League season found that the highest number of meters covered at maximum speed occurred in mid-season [15]. Furthermore, during the mid-season, players covered a higher distance per minute (106 m·min^−1^) compared to the end season (103 m·min^−1^; [16]). However, these studies have only analyzed the match physical demands; few studies have analyzed the TL evolution across the season. Only two studies reported that weekly TL decreased as the season progressed from mid-season toward the end of the season, highlighting the need to modulate the TL over the season [12,13].

The aforementioned studies have analyzed the influence of previous match outcome and seasonal period on weekly TL. However, there is limited scientific literature regarding the impact of performance streaks in team sports, a factor that considers match outcomes over time. Related to performance streaks, previous studies have examined coaches’ behavior during losing streaks (i.e., three consecutive defeats), highlighting their focus on providing support and improving players’ morale [17]. Other studies have explored the relationship between streaks and batting patterns in major league baseball [18]. However, limited data exist on performance streaks in soccer, and no prior research has investigated their relationship with weekly TL. Considering the gap about the influence of performance streaks (i.e., a variable to identify team performance linked to their persistency over time) on soccer, this study tried to examine whether the performance streaks reached during the competitive period of a professional male soccer team influenced weekly TL.

Therefore, the aim of this study was threefold: (i) to analyze the influence of previous match outcome on subsequent weekly TL; (ii) to examine whether accumulated weekly TL varies throughout the season; and (iii) to investigate the influence of performance streaks during competition on subsequent weekly TL. Based on findings from previous studies, the following hypotheses were proposed: (i) weekly TL following a win was lower when compared to a loss or draw; (ii) weekly TL decreased as the season progressed; and (iii) concerning performance streaks, although there are no previous scientific studies related to this issue, we expected that weekly TL after high-performance streaks was higher compared to low- or medium-performance streaks.

## 2. Materials and Methods

### 2.1. Study Design

This study employed a retrospective and longitudinal design to examine the differences in weekly TL according to previous match outcome, seasonal periods, and performance streaks in a professional male soccer team. All training sessions during the 2023/24 competitive season were monitored (from 3 September 2023 to 5 May 2024). To ensure consistency in comparisons and reduce variability, only weeks with one official match were included in the analysis [19]. In addition, when individual TL was not available for the entire week, weekly data from that player were excluded from the analysis. As a result, data from 34 weeks, a total of 171 training sessions, and 34 official soccer matches during the in-season period were considered. All soccer players who participated in the team during the whole season were considered in the study. All the training sessions were performed between 10 am and 12 pm and conducted on regular soccer pitches. Training data were collected daily to analyze differences in external load variables. Match outcomes from the previous week and seasonal periods were considered. Additionally, the performance streaks (i.e., points per match awarded) of the soccer team during the last five matches were also included.

### 2.2. Participants

A total of 21 professional male soccer players (age: 25.7 ± 2.7 years; height: 180.7 ± 7.3 cm; weight: 75.4 ± 6.9 kg) voluntarily participated in this study. All the players were recruited from the same team, which competed in the Second Federation championship (Fourth Spanish Division) during the 2023/24 season. From the team, five central defenders, five wide defenders, four central midfielders, four wide midfielders, and three forwards were included in the analysis. Goalkeepers were included in training sessions but excluded from the analysis due to their specific role. During the experimental period, players completed five weekly training sessions, each lasting an average of 110 min, and participated in one official match per week. This training routine remained consistent throughout the study. A total of 3073 individual observations belonging to training sessions were recorded. Although all the data were collected as a condition of employment, in which players are monitored daily over the competitive season, approval for the study from the soccer club was also obtained. Participants were fully informed about the study’s aims and potential risks before providing written informed consent. The study adhered to the ethical principles of the Declaration of Helsinki (2013) and received approval from the University of Extremadura’s Ethics Committee (protocol number: 33/2024).

### 2.3. Instruments

External workload during training sessions was measured using GPS devices (Catapult Sports, Melbourne, Australia). Each player consistently used the same device throughout the season, positioned at the center of the upper back [20]. The GPS device incorporates a tri-axial accelerometer, gyroscope, and magnetometer, all operating at a sampling rate of 100 Hz, while the GPS module functions at 10 Hz [21]. To ensure accurate satellite signal acquisition and synchronization, devices were activated 15 min before each training session or match, following the manufacturer’s guidelines. Previous studies have confirmed the high reliability of similar Catapult devices [22,23].

### 2.4. Variables

#### 2.4.1. Weekly External Load

The mean of each external load variable during all training sessions of the week was calculated for any players who competed in the match regardless of the minutes played and who had participated in all training sessions during the week of the match. Then, the sum of each variable during all training sessions of the week (excluding official matches) was also calculated per player, thus providing the weekly load for each variable [6,19]. Thus, external workload was assessed using the following variables: total distance covered (TD, meters); medium-speed running (MSR, 10.8–18.0 km·h^−1^); high-speed running (HSR, >18.0 km·h^−1^); very high-speed running (VHSR, 18.0–25.2 km·h^−1^); sprinting-speed running (sprint, >25.2 km·h^−1^); accelerations (ACC, >4 m·s^−2^); decelerations (DEC, <−4 m·s^−2^); and player load (PL, meters), calculated from accelerometer data across vertical, anterior-posterior, and medial-lateral axes. All variables were expressed as mean values per session.

#### 2.4.2. Latest Match Outcome

Match outcome from the previous match was categorized as follows: loss (i.e., when the previous match was lost; *n* = 885 observations), draw (i.e., when the previous match ended in a tie; *n* = 1059 observations), and Win (i.e., when the previous match was won; *n* = 1030 observations). For the calculation of this variable, the first training week was not considered because there was not any previous official match.

#### 2.4.3. Seasonal Period

Similar to previous studies [13,24], the whole season was split into different blocks of weeks to allow for an easier interpretation of the data. Specifically, according to the number of months per season of the competition, the season was split into eight periods: weeks 1–4 (i.e., month 1, September; *n* = 386 observations), weeks 5–9 (i.e., month 2, October, *n* = 438 observations), weeks 10–13 (i.e., month 3, November, *n* = 405 observations), weeks 14–16 (i.e., month 4, December, *n* = 275 observations), weeks 17–20 (i.e., month 5, January, *n* = 344 observations), weeks 21–24 (i.e., month 6, February, *n* = 303 observations), weeks 25–29 (i.e., month 7, March, *n* = 498 observations), and weeks 30–34 (i.e., month 8, April and May, *n* = 424 observations).

#### 2.4.4. Performance Streaks

To measure the influence of match outcome over time (i.e., performance streak) on subsequent weekly TL, the points per match awarded across the five last matches were considered. Then, the number of total points awarded was classified by terciles into three groups: low performance (i.e., training week after the team earned 5 points or less in the last five matches, *n* = 1011 observations), medium performance (i.e., training week after the team earned between 5 and 7 points in the last five matches, *n* = 734 observations), and high performance (i.e., training week after the team earned 9 or more points in the last five matches, *n* = 837 observations). This variable allows for knowing the weekly TL distribution after different performance streaks of the team. Similarly to the “latest match outcome” variable, until the sixth training week there were no data for this variable.

### 2.5. Data Analysis

All statistical analyses were performed using RStudio (version 2024.12.0 + 467) and the *dplyr* and *tidyr* packages for data manipulation and statistical calculations. Considering the characteristics of the sample, organized hierarchically in groups and with a longitudinal structure, we considered that the best procedure to analyze the data was through linear mixed models (LMMs).

Thus, LMMs were conducted to examine the influence of match outcome, seasonal period, and performance streaks on weekly external load using the *lme4* package [25]. A hierarchical level structure with the soccer player as the nesting unit of the training observations was considered for the analysis. Therefore, a two-level hierarchy was modeled for the analysis. The total weekly external load was calculated by the sum of daily averages to obtain a weekly total load for each variable. Then, the sum of external load variables (i.e., TD, MSR, HSR, VHSR, sprint, ACC, DEC, and PL) was included as dependent variables in the models, and match outcome (i.e., lose, draw, and win), seasonal period (i.e., September, October, November, December, January, February, March, April, and May), and performance streaks (i.e., low performance, medium performance, and high performance) were the independent variables included as fixed effects. The variable soccer player was considered as the random effect in the analysis. Values were represented as coefficients and standard error (Coeff ± SE). Then, a general multilevel modeling strategy was applied where fixed and random effects in different steps were included [26,27]. Finally, to assess statistical significance, Cohen’s effect sizes (ES; [28]) were also calculated to quantify the magnitude of differences observed in between-week comparisons. The thresholds for effect size interpretation were set as follows: trivial (<0.20), small (0.20–0.59), moderate (0.60–1.19), large (1.20–1.99), very large (2.00–3.99), and extremely large (>4.00).

## 3. Results

### 3.1. The Influence of Latest Match Outcome on Weekly External Load

**Figure 1:**
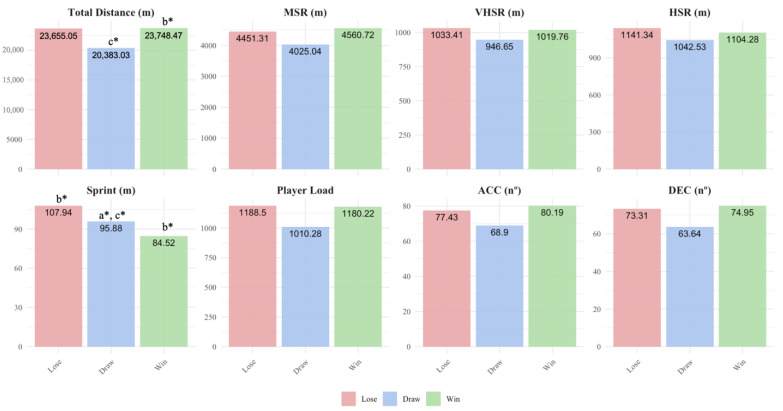
Weekly training load differences according to latest match outcome. Note: TD = total distance; MSR = medium-speed running; HSR = high-speed running; VHSR = very high-speed running; sprint = sprinting speed running; PL = player load; ACC = accelerations; DEC = decelerations; a = significant differences respect to lose; b = significant differences respect to draw; c = significant differences respect to win; * *p* < 0.05.

Figure 1 depicts the differences in subsequent weekly accumulated external load according to previous match outcome. Team covered significantly less TD during the week after draw than after win (*p* < 0.05), and less PL after draw than after lose (*p* < 0.05) and after win (*p* < 0.05). Although no significant differences were found, the team covered less MSR, VHSR, HSR, ACC, and DEC after draw than after lose and win.

### 3.2. The Influence of Seasonal Period on Weekly External Load

**Figure 2:**
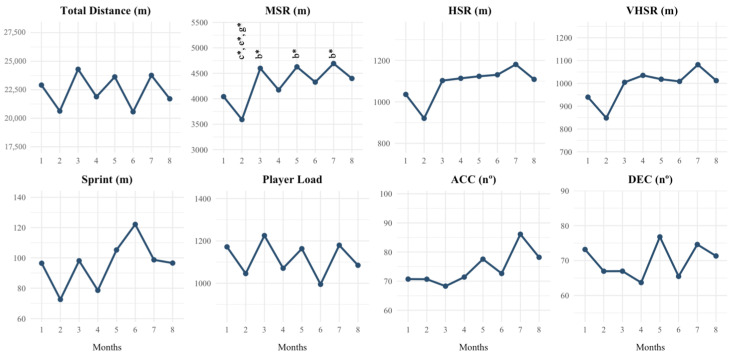
Weekly training load evolution across seasonal periods. Note: TD = total distance; MSR = medium-speed running; HSR = high-speed running; VHSR = very high-speed running; sprint = sprinting speed running; PL = player load; ACC = accelerations; DEC = decelerations; b = significant differences with period 2; c = significant differences with period 3; e = significant differences with period 5; g = significant differences with period 7; * *p* < 0.05.

Figure 2 shows the differences across seasonal periods in weekly accumulated external load. Team covered significantly less MSR during month 2 (i.e., October) compared to month 3 (i.e., November; *p* < 0.05), month 5 (i.e., January, *p* < 0.05), and month 7 (i.e., March, *p* < 0.05). No significant differences were found between different months on TD, HSR, VHSR, sprint distance, PL, ACC, and DEC.

### 3.3. The Influence of Performance Streaks on Weekly External Load

The differences in weekly accumulated external load after the performance streaks are presented in Table 1. After medium-performance streaks, team covered significantly higher TD compared to high-performance streaks (*p* < 0.05; ES = 1.11), and low-performance streaks (*p* < 0.01; ES = 1.36); higher MSR than high-performance streaks (*p* < 0.05; ES = 1.00), and low-performance streaks (*p* < 0.01; ES = 1.55); higher HSR and VHSR than low-performance streaks (*p* < 0.05; ES = 1.09 and ES = 0.99, respectively); higher sprint distance than high-performance streaks (*p* < 0.05; ES = 1.10), and low-performance streaks (*p* < 0.05; ES = 1.29); higher PL than high-performance streaks (*p* < 0.05; ES = 1.13), and low-performance streaks (*p* < 0.01; ES = 1.33); higher ACC than high-performance streaks (*p* < 0.05; ES = 1.11), and low-performance streaks (*p* < 0.01; ES = 1.50); and higher DEC than high-performance streaks (*p* < 0.05; ES = 0.95), and low-performance streaks (*p* < 0.01; ES = 1.36).

## 4. Discussion

The aim of this study was threefold: (i) to analyze the influence of previous match outcome on subsequent weekly TL; (ii) to examine whether accumulated weekly TL varies throughout the season; and (iii) to investigate the influence of performance streaks got during competition on subsequent weekly TL. This research provides useful knowledge to the scarce published literature about the influence of match-related contextual variables on the subsequent weekly TL and its variation throughout the season on a professional male soccer team. The main findings of this study were that (a) although no significant differences were found, weekly TL was lower after drawn matches; concretely, the team covered significantly lower TD during the week after draw than after win; (b) there was not a clear trend in the evolution of TL; however, most of the variables decreased as the season progressed; and (c) team accumulated higher weekly TL after medium-performance streaks. In general, the results were not in line with the expectations, since these findings could only confirm the second hypothesis related to the weekly TL evolution throughout the season.

Previous studies evaluating the influence of match outcome on subsequent weekly TL reported that weekly accumulated TL following a win was lower compared to a lost or drawn match [11,12,13]. Contrary to our hypothesis, this study did not find significant differences on subsequent TL when the previous match outcome was considered. Only the accumulated TD and PL were significantly lower during the week after drawn matches than after won matches. Therefore, these findings show that match-related contextual variables seem to slightly affect weekly external TL. Although our results did not replicate those previously reported in the literature, the coaching strategies depend on each team and player’s needs [12,29]. These results could also be explained by different mental load consequences on training weeks. Concretely, after lost matches, mental load could be greater on subsequent training weeks, possibly involving greater training demands for soccer players [30]. For that reason, future research should investigate the effect of previous match outcome on both external, internal, and mental TL in professional soccer for getting a holistic approach of TL.

Concerning our second hypothesis, we expected that weekly TL decreased as the season progressed. Although significant differences were not found in accumulated weekly TL, in general, training demands were lower as the season progressed. Only the MSR was significantly less during month 2 compared with various months. These findings could be explained based on previous studies about the evolution of match physical demands throughout the soccer season, where the physical performance of soccer players has seasonal changes [14,15]. Concretely, research on match running performance evolution has reported that higher values have been reached for soccer players at mid-season and then decrease at end-season [24,31]. However, there are only two studies that analyzed the evolution of weekly TL in a professional soccer setting, showing a decrease in TL at end-season [12,13]. Our results are similar to those provided in previous studies. This suggests that a general decrease in accumulated weekly TL throughout the season might have been related to the influence of chronic fatigue on soccer players along the season [32] or changes in training activities implemented by the technical staff to optimize the player performance (i.e., tapering or recovery training sessions) [13,33]. So, when planning training sessions, soccer coaches need to consider the period of the season to optimize soccer players performance according to fitness level and avoid overreaching injuries [34].

The term “performance streak” has commonly been used to describe a prolonged outcome in sport performance [35]. This term is used to analyze the performance of professional athletes over time. However, the influence of performance streaks on subsequent weekly TL has not been investigated so far. Our results showed that the team accumulated higher weekly TL after medium-performance streaks. In addition, high-speed running and sprinting speed running distances, ACC, and DEC were higher during these weeks. A possible explanation for these results might be that after medium-performance streaks, the coaching strategies aimed to improve the results of the next matches by higher training demands. Although there is not any previous research based on this issue, match analysis has shown that top-ranked teams covered greater distances and high-speed running distances in matches [36,37,38]. These findings suggest that reaching medium-performance streaks could be an important aim to get success at the end-season, and technical staff would involve higher training demands for those weeks with the aim to once again get a new win in the next matches [29]. Conversely, the lowest accumulated TL reached after low-performance streaks could be explained by the opponent level from the previous matches. Maybe the opponent teams during low-performance streaks were top- or medium-ranked teams, and research has reported that the highest values of high-speed running distances were observed after playing top-ranked teams [38], which suggests that the degree of neuromuscular fatigue may also be higher during the following week [29]. Then, coaching strategies applied could aim to decrease training volume for those weeks.

### Limitations, Future Directions and Practical Applications

Concerning the limitations of the present research, some issues should be acknowledged: (i) no internal load variables (i.e., heart rate or subjective perceived exertion) were assessed; future studies should include these measures to get a holistic and more comprehensive approach to TL and physiological adaptations of soccer players over the season; (ii) only one soccer team was involved in the study, which makes it difficult to generalize the results. In addition, although the soccer players involved were professional, specific characteristics of the Fourth Spanish Division could make it difficult to extrapolate the results to other professional or elite contexts; (iii) mental load and mental fatigue-related variables were not included in the analysis. Some studies have reported that latest match outcome influence subsequent mental training demands and mental fatigue, which could influence on subsequent physical performance of soccer players [30], especially after lost matches or low-performance streaks; (iv) further studies should introduce the relationship between previous match physical demands and subsequent weekly or daily TL in order to describe the influence of fatigue from match on subsequent external load day by day and obtain a better and more precise management on TL during daily training sessions; and (v) finally, other match-related contextual variables such as match venue or opponent quality should be analyzed in further research.

On the other hand, the findings of this study may have great practical implications, which could help coaches and practitioners to better understand weekly external load in a soccer team competing in the Fourth Spanish Division. Specifically, strength and conditioning coaches should pay attention to TL management after different match outcomes and performance streaks to optimize players’ recovery strategies. For instance, after lost matches, technical staff should not significantly increase weekly TL since players will try to perform their maximum efforts during the next week to get a win in the next match or improve the performance. In addition, practitioners should reduce the volume of the training sessions after high- or medium-performance streaks. Specifically, identifying the weeks with higher training demands is crucial for effective load management and preventing injury risk from overreaching.

## 5. Conclusions

To our knowledge, this is the first study to date that has examined the influence of performance streaks on subsequent weekly TL in a professional male soccer team. The main results showed that the team accumulated higher weekly TL after medium-performance streaks. On the contrary, weekly TL was lower after drawn matches; concretely, the team covered significantly lower TD during the week after draw than after win. Finally, although there was not a clear trend on evolution on TL, most of the variables decreased as the season progressed. Therefore, the present study showed that match-related contextual variables seem to slightly affect weekly external TL.

## Figures and Tables

**Table 1:** Differences in weekly external load based on performance streaks.

Variables	High Performance	Medium Performance	Low Performance	*p*	Between Weeks Comparison (ES)		
	Coeff (SE)	Coeff (SE)	Coeff (SE)		HP vs. MP	HP vs. LP	MP vs. LP
TD (m)	21,637 (1250)	26,025 (1490)	20,665 (1140)	a *, c **	−1.11 (−2.18, −0.05)	0.25 (−0.64, 1.13)	1.36 (0.31, 2.41)
MSR (m)	4342 (217)	5025 (259)	3962 (198)	a *, c **	−1.00 (−2.05, 0.06)	0.56 (−0.34, 1.45)	1.55 (0.48, 2.62)
HSR (m)	1096 (73.9)	1254 (88.3)	1001 (67.4)	c *	−0.68 (−1.71, 0.35)	0.41 (−0.48, 1.30)	1.09 (0.06, 2.11)
VHSR (m)	1008 (63.9)	1120 (76.4)	920 (58.4)	c *	−0.56 (−1.58, 0.47)	0.43 (−0.46, 1.32)	0.99 (−0.03, 2.01)
Sprint (m)	88.3 (13.1)	134.1 (15.7)	80.7 (12.0)	a *, c *	−1.10 (−2.16, −0.04)	0.18 (−0.70, 1.07)	1.29 (0.24, 2.33)
PL	1069 (63.7)	1297 (76.1)	1029 (58.1)	a *, c **	−1.13 (−2.20, −0.07)	0.20 (−0.69, 1.08)	1.33 (0.28, 2.38)
ACC (n°.)	73.7 (4.95)	91.0 (5.91)	67.6 (4.52)	a *, c **	−1.11 (−2.17, −0.05)	0.39 (−0.50, 1.28)	1.50 (0.43, 2.57)
DEC (n°.)	68.5 (4.77)	82.7 (5.70)	62.3 (4.35)	a *, c **	−0.95 (−2.00, 0.10)	0.41 (−0.48, 1.30)	1.36 (0.31, 2.41)

Note: Coeff = coefficient; SE = standard error; ES = effect size; HP = high performance; MP = medium performance; LP = low performance; TD = total distance; MSR = medium speed running; HSR = high speed running; VHSR = very high-speed running; sprint = sprinting speed running; PL = player load; ACC = accelerations; DEC = decelerations; a = significant differences between high and medium performance; c = significant differences between medium and low performance; * *p* < 0.05; ** *p* < 0.01.

## Data Availability

The data are available upon request to the corresponding author.

## Abbreviations

The following abbreviations are used in this manuscript:

TL	Training load
TD	Total distance
MSR	Medium-speed running
HSR	High-speed running
VHSR	Very high-speed running
Sprint	Sprinting speed running
ACC	Accelerations
DEC	Decelerations
LMM	Linear mixed models
GPS	Global positioning system
ES	Effect size
